# On the chordae structure and dynamic behaviour of the mitral valve

**DOI:** 10.1093/imamat/hxy035

**Published:** 2018-08-30

**Authors:** Liuyang Feng, Nan Qi, Hao Gao, Wei Sun, Mariano Vazquez, Boyce E Griffith, Xiaoyu Luo

**Affiliations:** 1 School of Mathematics and Statistics, University of Glasgow, UK; 2 Institute of Marine Science and Technology, Shandong University, Shandong, China and School of Mathematics and Statistics, University of Glasgow, UK; 4 Wallace H Coulter Department of Biomedical Engineering, Georgia Institute of Technology, Atlanta, GA, USA; 5 Barcelona Supercomputing Centre, IIIA-CSIC, Spain; 6 Departments of Mathematics, Applied Physical Sciences, and Biomedical Engineering and McAllister Heart Institute, University of North Carolina, Chapel Hill, NC, USA

**Keywords:** mitral valve, chordae tendineae, fluid–structure interaction, immersed boundary method, finite element method

## Abstract

We develop a fluid–structure interaction (FSI) model of the mitral valve (MV) that uses an anatomically and physiologically realistic description of the MV leaflets and chordae tendineae. Three different chordae models—complex, ‘pseudo-fibre’ and simplified chordae—are compared to determine how different chordae representations affect the dynamics of the MV. The leaflets and chordae are modelled as fibre-reinforced hyperelastic materials, and FSI is modelled using an immersed boundary–finite element method. The MV model is first verified under static boundary conditions against the commercial finite element software ABAQUS and then used to simulate MV dynamics under physiological pressure conditions. Interesting flow patterns and vortex formulation are observed in all three cases. To quantify the highly complex system behaviour resulting from FSI, an energy budget analysis of the coupled MV FSI model is performed. Results show that the complex and pseudo-fibre chordae models yield good valve closure during systole but that the simplified chordae model leads to poorer leaflet coaptation and an unrealistic bulge in the anterior leaflet belly. An energy budget analysis shows that the MV models with complex and pseudo-fibre chordae have similar energy distribution patterns but the MV model with the simplified chordae consumes more energy, especially during valve closing and opening. We find that the complex chordae and pseudo-fibre chordae have similar impact on the overall MV function but that the simplified chordae representation is less accurate. Because a pseudo-fibre chordal structure is easier to construct and less computationally intensive, it may be a good candidate for modelling MV dynamics or interaction between the MV and heart in patient-specific applications.

## 1. Introduction

Mitral valve (MV) dysfunction, including MV stenosis, prolapse and regurgitation, is one of the most common valvular heart diseases and hence has attracted significant research interest. Computational modelling of human MV function (Gao *et al.*, 2017b) can improve our understanding of MV biomechanics (Kunzelman *et al.*, 1997, 1998), which is important for improving surgical procedures (Cochran & Kunzelman, 1998; Reimink *et al.*, 1996) and medical therapies (Kheradvar *et al.*, 2015). However, because of the challenges of modelling the highly complex MV structure, its deformation and its interaction with the left ventricle, only limited progress in multi-physics modelling of the MV has been made to date (Einstein *et al.*, 2010; Gao *et al.*, 2017b; Sun *et al.*, 2014).

Approaches to modelling the MV often fall into two categories: structural analysis and fluid–structure interaction (FSI) analysis. The former is simpler and focuses on MV deformation in its fully closed state. The latter focuses on the whole cardiac cycle and is more computationally demanding but provides a more complete description of valvular function. Both approaches have been used to study MV dynamics (Einstein *et al.*, 2010; Gao *et al.*, 2014a; Kunzelman *et al.*, 1993, 1997; Kunzelman & Cochran, 1992; Luo *et al.*, 2012; Ma *et al.*, 2013; Watton *et al.*, 2008). For a recent review of different MV modelling strategies, the reader is referred to Gao *et al.* (2017b). In this study, we use an immersed boundary–finite element (IB/FE) method (Griffith & Luo, 2017) to develop a dynamic MV FSI model.

The chordae tendineae in the MV apparatus, which connect the papillary muscle to MV leaflets, play an important role in MV function, especially in the systolic phase to prevent the leaflets prolapse. Studies have shown that chordae structure has a substantial impact on MV function (David *et al.*, 1984; Espino *et al.*, 2005; Reimink *et al.*, 1996). For example, David *et al.* (1984) found that to repair chronic MV regurgitation, post-operative left ventricular function benefits from preserving the chordae structure and papillary muscles when replacing the MV. Espino *et al.* (2005) also confirmed that anterior leaflet marginal chordae as well as commissural chordae are vital for MV competence. However, few studies have investigated the difference in MV function between different chordae structures with FSI. In our previous studies, we used various simplified chordae structures, including discrete elastic ‘pseudo-fibre’ models (Luo *et al.*, 2012; Ma *et al.*, 2013; Watton *et al.*, 2008) and fully 3D volumetric models (Gao *et al.*, 2017a, 2014a). However, those simplified chordae structures did not distinguish the marginal, basal and strut chordae. As a result, our modelled anterior leaflet bulged towards the left atrium, which is a common artefact of many existing MV models.

This study aims to overcome these defects and to investigate how different chordae representations affect valvular function. To do so, we use a physiologically detailed description of the MV leaflets and chordae structure based on multi-slice computed tomography (CT) image data (Wang & Sun, 2013). In addition, we consider three different chordae structure representations: complex, pseudo-fibre and simplified. The complex model uses a fully three-dimensional chordae structure based on the CT images, in which the marginal, basal and strut chordae are all included. The pseudo-fibre model is based on the same CT data but represented using thin elastic fibres, as in a more conventional IB approach (Ma *et al.*, 2013). The simplified chordae structure is based on our previous idealized chordae descriptions (Gao *et al.*, 2014a), in which the chordae are also modelled using a fully three-dimensional volumetric solid mesh. In all these cases, anisotropic material laws are used for both the MV leaflets and the chordae.

The paper is organized as follows: Sections 2 and 3 provide details of the IB/FE method and material properties. Section 4 describes verification studies of our IB/FE model against the commercial FE package ABAQUS (https://www.3ds.com/products-services/simulia/products/abaqus/) in steady-state conditions. Sections 5 and 6 provide implementation details and describe results for FSI simulations. We compare the MV model behaviour with different chordae structures and comparisons of MV orifice area during systolic contraction of the left ventricle (LV), and fluid flow patterns during diastolic filling of the LV phase are made among those three chordae structures. We also analyse energy budget for the fully coupled MV-flow model including detailed energy distribution and its dissipation. Section 7 discusses these results and provides a concluding discussion.

## 2. Methodology

### 2.1 IB/FE formulation

In the IB/FE formulation of FSI used in this study (Griffith & Luo, 2017), the structure elasticity and deformation are described in Lagrangian coordinates, and the pressure, velocity and incompressibility of the coupled fluid–structure system are described in Eulerian coordinates. Let $\varOmega \subset \mathbb{R}^3$ denote the fixed physical domain occupied by the coupled fluid–structure system, and let $\varOmega ^{\textrm{s}}_0\subset \varOmega $ be the Lagrangian reference coordinate domain for structure. $ \mathbf x \in U$ are fixed Cartesian physical coordinates, $ \mathbf X \in \varOmega ^{\textrm{s}}_0$ are the fixed Lagrangian reference coordinates of the structure and ${\boldsymbol{\chi}} ( \mathbf X,t) \in U$ is the current position of material point $ \mathbf X$ at time $t$. Thus, the region occupied by structure at time $t$ is $\varOmega ^{\textrm{s}}_t =  \boldsymbol{\chi} (\varOmega ^{\textrm{s}}_0,t)$, and $\varOmega ^{\textrm{f}}_t = \varOmega \backslash \varOmega ^{\textrm{s}}_t $ is the region occupied by the fluid at time $t$.

The Cauchy stress tensor is divided into the following two parts,

(1) \begin{equation*} {\boldsymbol{\sigma}}(\mathbf{x},\textit{t})= \begin{cases} {\boldsymbol{\sigma}}^{\textrm{f}}(\mathbf{x},\textit{t})+ {\boldsymbol{\sigma}}^{\textrm{e}}(\mathbf{x},\textit{t}), & \mathbf{x} \in \varOmega^{\textrm{s}}_{\textit{t}},\\{\boldsymbol{\sigma}}^{\textrm{f}}(\mathbf{x},\textit{t}), & \textrm{otherwise}. \end{cases} \end{equation*}

Here, ${\boldsymbol{\sigma}} ^{\textrm{f}}(\mathbf{x},\textit{t})$ is the Cauchy stress tensor for a viscous incompressible fluid,

(2) \begin{equation*} {\boldsymbol{\sigma}}^{\textrm{f}}(\mathbf{x},\textit{t})= -p\mathbb{I}+\mu(\nabla\mathbf{u}+\nabla\mathbf{u}^{\textit{T}}), \end{equation*}

in which $p$ and $\mathbf{u}$ are the material pressure and velocity, and $\mu $ is the dynamic viscosity of the fluid. The Cauchy elastic stress tensor ${\boldsymbol{\sigma}} ^{\textrm{e}}(\mathbf{x},\textit{t})$ can be related to the first Piola–Kirchhoff stress tensor $\mathbb{P}^{\textrm{e}}$ using Nanson’s formula,

(3) \begin{equation*} {\int_{\partial V}} \mathbb{P}^{\textrm{e}} (\mathbf{X},\textit{t})\mathbf{N}\, \textrm{d}\textit{A}(\mathbf{X}) = \int_{\partial{\boldsymbol{\chi}}(\textit{V,t})} {\boldsymbol{\sigma}}^{\textrm{e}} (\mathbf{x},\textit{t})\mathbf{n}\,\textrm{d}\textit{a}(\mathbf{x}), \end{equation*}

in which $V\subset \varOmega ^{\textrm{s}}_0$ is an arbitrary Lagrangian subregion, $ \mathbf N$ is the outward unit vector along $\partial V$ and $ \mathbf n$ is the outward unit vector along $\partial  \boldsymbol{\chi} (V,t) $.

The equations for the IB/FE formulation of FSI used in this study are (Griffith & Luo, 2017)

(4) \begin{equation*} \rho \left(\frac{\partial \mathbf u(\mathbf x,t)}{\partial t }+\mathbf u(\mathbf x,t)\cdot \nabla \mathbf u(\mathbf x,t)\right)=-\nabla p(\mathbf x,t)+\mu\nabla^2 \mathbf u(\mathbf x,t)+\mathbf f(\mathbf x,t), \end{equation*}

(5) \begin{equation*} \nabla\cdot\mathbf u(\mathbf x,t)=0, \end{equation*}

(6) \begin{equation*} \mathbf f(\mathbf x,t)=\int_{\varOmega^{\textrm{s}}_0} \mathbf F(\mathbf X,t)\,\delta(\mathbf x-{\boldsymbol{\chi}}(\mathbf X,t))\,\textrm{d}\mathbf X, \end{equation*}

(7) \begin{equation*} \int_{\varOmega^{\textrm{s}}_0}\mathbf F(\mathbf X,t)\cdot\mathbf V(\mathbf X)\,\textrm{d}\mathbf X=-\int_{\varOmega^{\textrm{s}}_0}\mathbb{P}^{\textrm{e}}(\mathbf X,t):\nabla_{\mathbf X}\mathbf V(\mathbf X)\,\textrm{d}\mathbf X ,\ \forall \mathbf V(\mathbf X), \end{equation*}

(8) \begin{equation*} \frac{\partial {\boldsymbol{\chi}}}{\partial t }(\mathbf X,t)=\mathbf u({\boldsymbol{\chi}}(\mathbf X,t),t)=\int_{\varOmega}\mathbf u(\mathbf x,t)\,\delta({\boldsymbol{\chi}}(\mathbf X,t)-\mathbf x)\,\textrm{d}\mathbf x, \end{equation*}

with $ \mathbf V( \mathbf X)$ an arbitrary Lagrangian test function. Equation (4) is the momentum equation for the coupled system, (5) is the incompressibility constraint and (6) and (8) are interaction equations that connect the Lagrangian and Eulerian coordinate systems, which use the delta function $\delta ( \mathbf x)$ to transfer quantities between the two frames. For problems discussed here, $\rho $ and $\mu $ are assumed constants throughout the system. Equation (6) relates the Lagrangian elastic force density $ \mathbf F( \mathbf X,t)$ and the corresponding Eulerian elastic force density $ \mathbf f( \mathbf x,t)$. Equation (8) implies that the no-slip condition is satisfied at the fluid–solid interface. We remark that although we used two separate set of meshes, one Eulerian and one Lagrangian, only the Eulerian momentum equation is solved for the coupled system, because the Eulerian momentum equation accounts for the momentum of the entire computational domain $\varOmega = \varOmega ^{\textrm{f}}_t \cup \varOmega ^{\textrm{s}}_t$. The applied forces ensure traction continuity at the fluid–solid interface.

### 2.2 Energy budget analysis

Now we introduce the energy budget analysis for the coupled FSI model. The energy balance of the FSI system can be written as

**Fig. 1.**
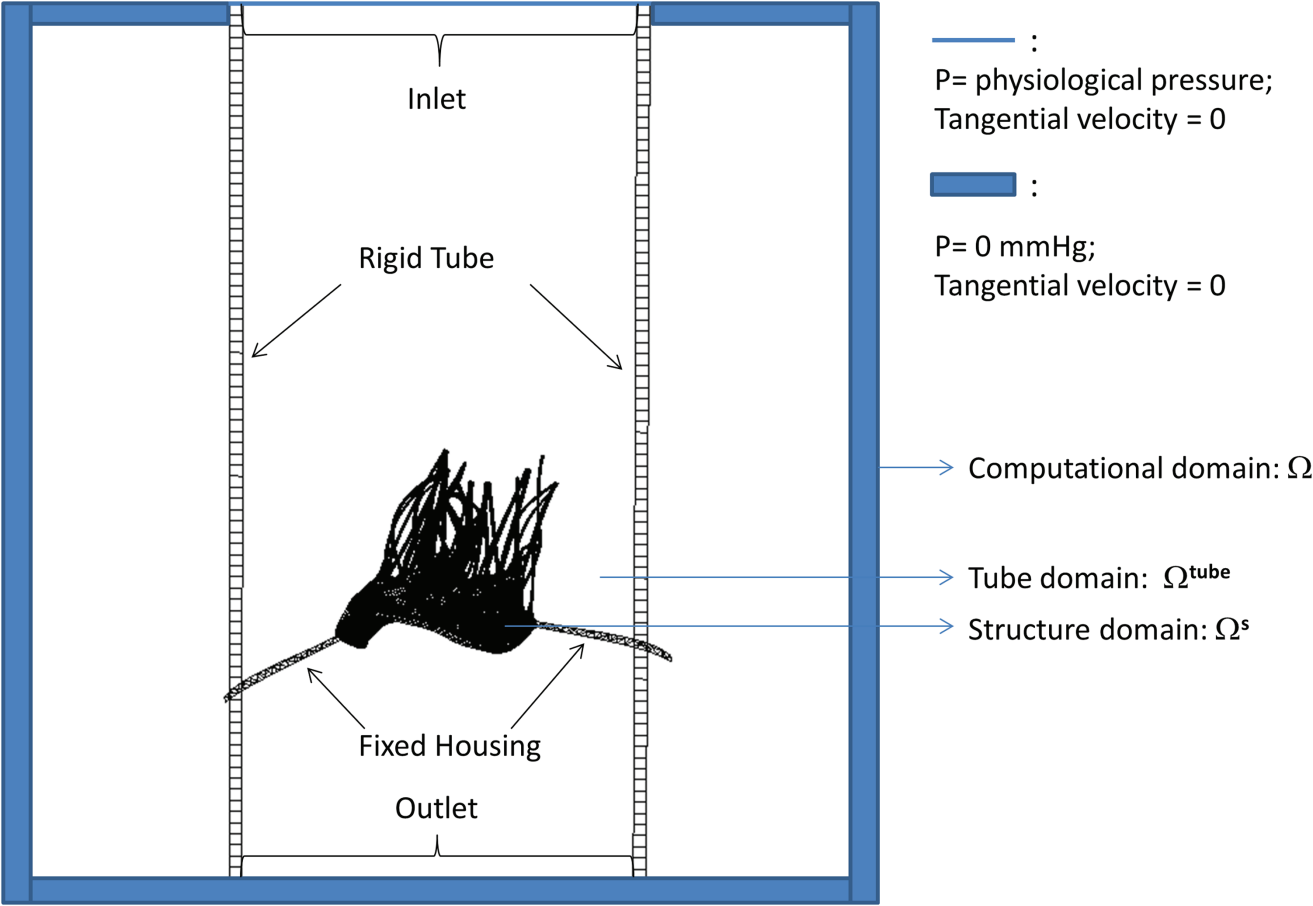
Schematic illustration of the MV-fluid coupled system.

(9) \begin{equation*} \int_{\varOmega^{\textrm{tube}}} \rho\left(\frac{\partial \mathbf u}{\partial t }\right)\cdot \mathbf u\, \textrm{d}\mathbf x + \int_{\varOmega^{\textrm{tube}}} \rho(\mathbf u\cdot \nabla)\mathbf u\cdot \mathbf u\, \textrm{d}\mathbf x=\int_{\varOmega^{\textrm{tube}}}\left(-\nabla p+\mu\nabla^2 \mathbf u\right)\cdot \mathbf u\, \textrm{d}\mathbf x +\int_{\varOmega^{\textrm{tube}}} \mathbf f\cdot \mathbf u\, \textrm{d}\mathbf x, \end{equation*}

in which $\varOmega ^{\textrm{tube}}$ is the interior of the stationary tube in which the MV is mounted (Fig. 1). Equation (9) consists of the change of the kinetic energy,

(10) \begin{equation*} \textrm{KE}=\int_{\varOmega^{\textrm{tube}}} \rho\left(\frac{\partial \mathbf u}{\partial t }\right)\cdot \mathbf u\ \textrm{d}\mathbf x=\frac{\mathrm d}{{\mathrm d} t} \left(\int_{\varOmega^{\textrm{tube}}} \frac{1}{2}\rho\mathbf u\cdot\mathbf u\, \textrm{d}\mathbf x \right), \end{equation*}

the kinetic energy flux of fluid on the boundary $\partial U$,

(11) \begin{equation*} \textrm{KF}=\int_{\varOmega^{\textrm{tube}}} \rho(\mathbf u\cdot \nabla)\mathbf u\cdot \mathbf u\, \textrm{d}\mathbf x=\int_{\partial U}\frac{1}{2}\rho(\mathbf u\cdot\mathbf u) \mathbf u\cdot\mathbf n\, \textrm{d}a, \end{equation*}

the rate of work by the applied pressure,

(12) \begin{equation*} \textrm{WP}=\int_{\varOmega^{\textrm{tube}}}\left(-\nabla p\right)\cdot \mathbf u\, \textrm{d}\mathbf x =\int_{\partial U} \left(-p\right)\mathbf n\cdot \mathbf u\, \textrm{d}a, \end{equation*}

the rate of energy dissipation,

(13) \begin{align*} \textrm{D}=\int_{\varOmega^{\textrm{tube}}}\mu\nabla^2 \mathbf u\cdot \mathbf u\, \textrm{d}\mathbf x=-\int_{\varOmega^{\textrm{tube}}}\mu (\nabla \mathbf u+\nabla \mathbf u ^{T}): \nabla \mathbf u\, \ \textrm{d}\mathbf x, \end{align*}

and the rate of change of elastic strain energy in the immersed structure,

(14) \begin{align*} \textrm{E}=\int_{\varOmega^{\textrm{tube}}} \mathbf f\cdot \mathbf u\, \textrm{d}\mathbf x =\int_{\varOmega^{\textrm{tube}}} \left(\int_{\varOmega^{\textrm{s}}_0} \mathbf F(\mathbf X)\delta(\mathbf x-{\boldsymbol{\chi}}(\mathbf X,t))\,\mathrm{d}\mathbf X\right)\cdot \mathbf u\, \textrm{d}\mathbf x \end{align*}

(15) \begin{align*} =\int_{\varOmega^{\textrm{tube}}} \left(\int_{\varOmega^{\textrm{s}}_0} \mathbf u(\mathbf x,t) \delta(\mathbf x-{\boldsymbol{\chi}}(\mathbf X,t))\, \textrm{d}\mathbf x\right)\cdot \mathbf F(\mathbf X)\, \textrm{d}\mathbf X \end{align*}

(16) \begin{align*} =\int_{\varOmega^{\textrm{s}}_0} \mathbf u({\boldsymbol{\chi}}(\mathbf X,t),t) \cdot \mathbf F(\mathbf X)\, \textrm{d}\mathbf X, \end{align*}

which can be rewritten as

(17) \begin{equation*} \textrm{E}=-\int_{\varOmega^{\textrm{s}}_0}\mathbb{P}^{\mathrm{e}}(\mathbf X,t):\nabla_{\mathbf X}\mathbf u({\boldsymbol{\chi}}(\mathbf X,t),t)\, \textrm{d}\mathbf X, \end{equation*}

with the help of (7).

**Fig. 2.**
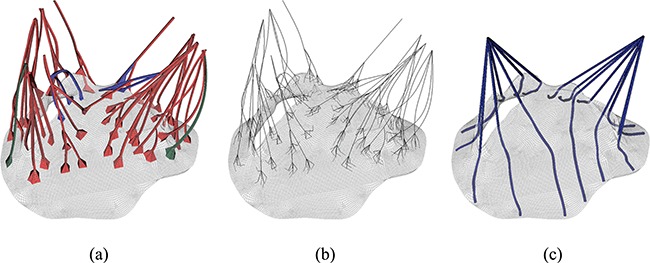
MV models with (a) complex chordae, (b) pseudo-fibre chordae and (c) simplified chordae. In (a) the struct chordae are coloured in red, the marginal chordae are coloured in blue and the basal chordae are coloured in green (see online version). In (b) and (c), only one chordae type is considered.

### 2.3 Spatial and temporal discretizations

A Cartesian grid is used to discretize the Eulerian domain $U$. We use a staggered grid to approximate the Eulerian velocity $ \mathbf u=(u_x,\ u_y,\ u_z)$ and Eulerian force density $ \mathbf f=(f_x,\ f_y,\ f_z)$. Standard second-order accurate finite difference approximations to the divergence, gradient and Laplace operators (Griffith, 2009) are used. The nonlinear advection terms in (4) are discretized using a version of the piecewise parabolic method (Colella & Woodward, 1984). We discretize the Lagrangian domain using the finite element method with trilinear ($Q^1$) basis functions for the displacement and resultant force. Additional details of the discretization can be found in Griffith & Luo (2017).

## 3. MV geometry and material properties

Our MV model includes a detailed MV anatomical geometry obtained from multi-slice CT images of a normal MV at mid-diastole (when the MV is open) obtained from a 61-year-old male patient (Wang & Sun, 2013). Three different models are built, as shown in Fig. 2. All three models have the same leaflets but different chordae representations. The first one, shown in Fig. 2(a), is referred to as the ‘complex’ model. It has a CT-derived chordal structure that includes the marginal, basal and strut chordae, with a fork-shaped geometry at chordae–leaflet connection to avoid stress concentrations. The chordae are modelled as fully three-dimensional solid bodies using a volumetric mesh discretization with a uniform cross-sectional area of 0.16 mm$^{2}$ (Gao *et al.*, 2014a). The second model, shown in Fig. 2(b), is based on the same CT scan data, but here the chordae are represented as one-dimensional elastic fibres, as in more conventional IB approaches (Ma *et al.*, 2013). The chordae follow the same structure as in the complex model, including all three types of chordae, and a fork-shaped geometry at the chordae–leaflet connection. In other words, the pseudo-fibres are the skeleton of the complex chordae derived from the CT scan data (Wang & Sun, 2013). We refer this as the ‘pseudo-fibre’ model. The third model, shown in Fig. 2(c), uses a simplified chordae structure, as in our previous work (Gao *et al.*, 2014a), which has a total of 16 evenly distributed chordae (6 on anterior leaflet and 10 on posterior leaflet) with the same uniform cross-sectional area of 0.16 mm$^2$. The anterolateral and posterolateral papillary muscle groups are represented by two chordae attachment movements obtained from the average of chordae attachment positions from the complex model. These chordae are also modelled as volumetric solids and run through the leaflets from the free leaflet edges to the annulus ring. Details can be found in Gao *et al.* (2014a). This is referred to as the ‘simplified’ chordae model.

**Fig. 3.**
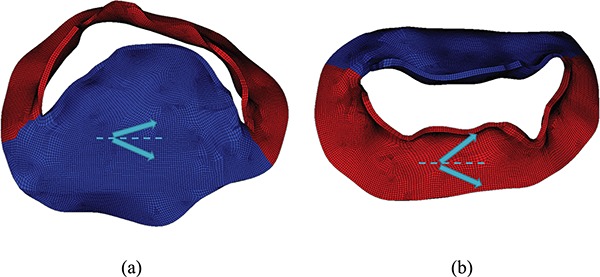
Two families of fibre directions shown by arrows on anterior, shown in (a) (blue online) and posterior, shown in (b) (red online) leaflet. Circumferential direction is shown by the dashed line.

An invariant-based, anisotropic constitutive model based on the formulation proposed by Gasser *et al.* (2006) and Holzapfel *et al.* (2000) is used to describe the MV leaflets:

(18) \begin{equation*} W^{\textrm{leaflet}}=C_1\{\exp[C_2(I_1 -3)]-1\}+\frac{k_1}{2k_2}\sum_{i=1}^2\left\{\exp{\left[k_2\left(I_{4i}^{\ast}-1\right)^2\right]}-1\right\}+\frac{\beta}{4}\log^2(I_3), \end{equation*}

in which $I_{1}=\textrm{trace}(\mathbb{F}^{T}\mathbb{F})$, $I_{3}=\textrm{det}(\mathbb{F}^{T} \mathbb{F})= J^{2}$ and $\mathbb{F} = \partial \chi / \partial \mathbf{X}$ is the deformation gradient tensor. Here, $\frac{\beta }{4}\log ^{2}(I_{3})$ is a volumetric energy that acts to penalize compressible deformations. We choose $\beta = \textrm{500 kPa}$ in our simulations. The modified fibre pseudo-invariants are $I_{4i}^{\ast }= \max $$[(\kappa I_{1} + (1-3 \kappa )I_{4i}),1]$ and $I_{4i}=\mathbf{e}_{i}\cdot (\mathbb{F}^{T} \mathbb{F}){\mathbf{e}_{i}}$, using the fibre directions $\mathbf{e}_{i}$ shown in Fig. 3(a and b). Two families of fibres are included here, whose directions as well as the parameter values are directly from biaxial experimental data for a healthy human mitral leaflets (Wang & Sun, 2013). The modified invariant $I^{\ast }_{4i}$ is defined to ensure that only extended fibres contribute to the strain energy. In particular, the fibres do not support compressive loads. $\kappa $ is a collagen fibre dispersion parameter, and $C_1$, $C_2$, $k_1$ and $k_2$ are the material parameters. The parameter values for the models are summarized in Table 1.

We define the modified elastic stress tensor $\mathbb{P}^{\textrm{e}}$ via

(19) \begin{equation*} \begin{aligned} \mathbb{P}^{\textrm{e}}_{\textrm{leaflet}}\!=&\, 2C_1C_2\exp[C_2(I_1 -3)]\mathbb{F}-2C_1C_2\exp[C_2(I_1 -3)]\mathbb{F}^{-T} \\ &\!+\sum_{i=1}^2 2k_1\kappa(I_{4i}^{\ast}-1)\!\exp{\!\left[k_2\!\left(I_{4i}^{\ast}-1\right)^2\right]}\mathbb{F}+\!\sum_{i=1}^2 2k_1(1-3\kappa)(I_{4i}^{\ast}-1)\exp{\!\Big[k_2\left(I_{4i}^{\ast}-1\right)^2\Big]}\mathbb{F}\mathbf e_i\otimes\mathbf e_i\\ &\!+\beta\log(I_3)\mathbb{F}^{-T}. \end{aligned} \end{equation*}

The term $2C_1C_2\exp [C_2(I_1 -3)]\mathbb{F}^{-T}$ is included to ensure $\mathbb{P}^{\textrm{e}}_{\textrm{leaflet}}$ is zero when $\mathbb{F}=\mathbb{I}$. This modification is found to reduce the magnitude of spurious volume loss caused by the pressure discontinuities across the structure–fluid interface (Gao *et al.*, 2014b).

**Table 1:** Material parameters of MV leaflets, from Wang & Sun (2013)

	$C_1$ (kPa)	$C_2$	$k_{1}$ (kPa)	$k_2$	$\kappa $
Anterior	0.12	13.67	11.01	84.85	0.08
Posterior	0.05	15.00	3.02	144.48	0.053

In all models of the chordae tendinae, the elasticity of the chordal material is described by

(20) \begin{equation*} W^{\textrm{chordae}}=C(I_1 -3)+\frac{\alpha_1}{2\alpha_2}\left\{\exp{[\alpha_2(I^{\ast}_4 -1)^2]}-1\right\}+\frac{\beta}{4}\log^2(I_3), \end{equation*}

in which $C$, $\alpha _1$ and $\alpha _2$ are material parameters whose values are summarized in Table 2, and $I_1$, $I_3$ and $I_4^{\ast }$ are similarly defined without dispersion, i.e. $\kappa =0$. The fibre direction for all the chordae tendineae is simply defined along its longitudinal direction. For the complex model, all the branches have a fibre direction defined by following the long axis at the centre of the cross-section. As in the leaflets, we use $\beta =\text{500 kPa}$. The resulting stress tensor is

(21) \begin{equation*} \mathbb{P}^{\textrm{e}}_{\textrm{chordae}}=2C\mathbb{F}-2C\mathbb{F}^{-T}+2\alpha_1\exp{\left[\alpha_2(I^{\ast}_4 -1)^2\right]}\mathbb{F}\mathbf e\otimes\mathbf e+\beta\log(I_3)\mathbb{F}^{-T}. \end{equation*}

**Table 2:** Material parameters of MV chordae

	$C$ (kPa)	$\alpha _1$ (kPa)	$\alpha _2$
Basal chordae	540	1446.2	22.09
Marginal chordae	540	200.48	33.83
Struct chordae	540	1446.2	22.09

Although the pseudo-fibre model describes each chord as a one-dimensional elastic fibre, the fibre model uses the same nonlinear material description as in (20), taking the cross-sectional area of the chords to be $0.04$ cm $\times $$0.04$ cm. The cross-sectional area is chosen so that the total force of the pseudo-fibres on the MV equals to the total force exerted by the solid chordae.

## 4. IB/FE model verification

Detailed IB/FE model verification studies are performed on a disc geometry and the MV model with complex chordae under static boundary conditions. We compare the results obtained by the IB/FE model in static conditions to a corresponding structure-only model built in ABAQUS. The models are shown to be in good agreement; additional details are provided in Appendices A and B.

## 5. Dynamic MV-fluid model implementation

In the coupled MV-fluid system, the MV is mounted on a fixed housing structure that is placed inside a rigid tube with length 16 cm and radius 3.5 cm, as shown in Fig. 1. The whole structure sits inside an 8.6 cm $\times $ 8.6 cm $\times $ 16 cm computational domain filled with viscous fluid of density 1 g$\cdot $cm$^{-3}$ and dynamic viscosity 0.04 g$\cdot $cm$^{-1}\cdot $s$^{-1}$. The grid sizes for both fluid and solid are chosen so that good agreement is achieved in the verification problems (see Appendix for details). For the structural discretization, we use the same grid size for the leaflets, but because of the different treatment of the chordae, the total elements/nodes are 106963/153336 for the complex model, 97093/116964 for the pseudo-fibre model and 75292/92910 for the simplified model. For the Eulerian mesh, we use a two-level block-structured adaptively refined grid with a refinement ratio 4 between the levels. The finest level has a grid of $N_x=130$, $N_y=130$ and $N_z=200$. Hence, the fluid mesh size is in the order of 0.07 cm $\times $ 0.07 cm $\times $ 0.08 cm. The time step size is chosen to be 5.0e-6 s.

**Fig. 4.**
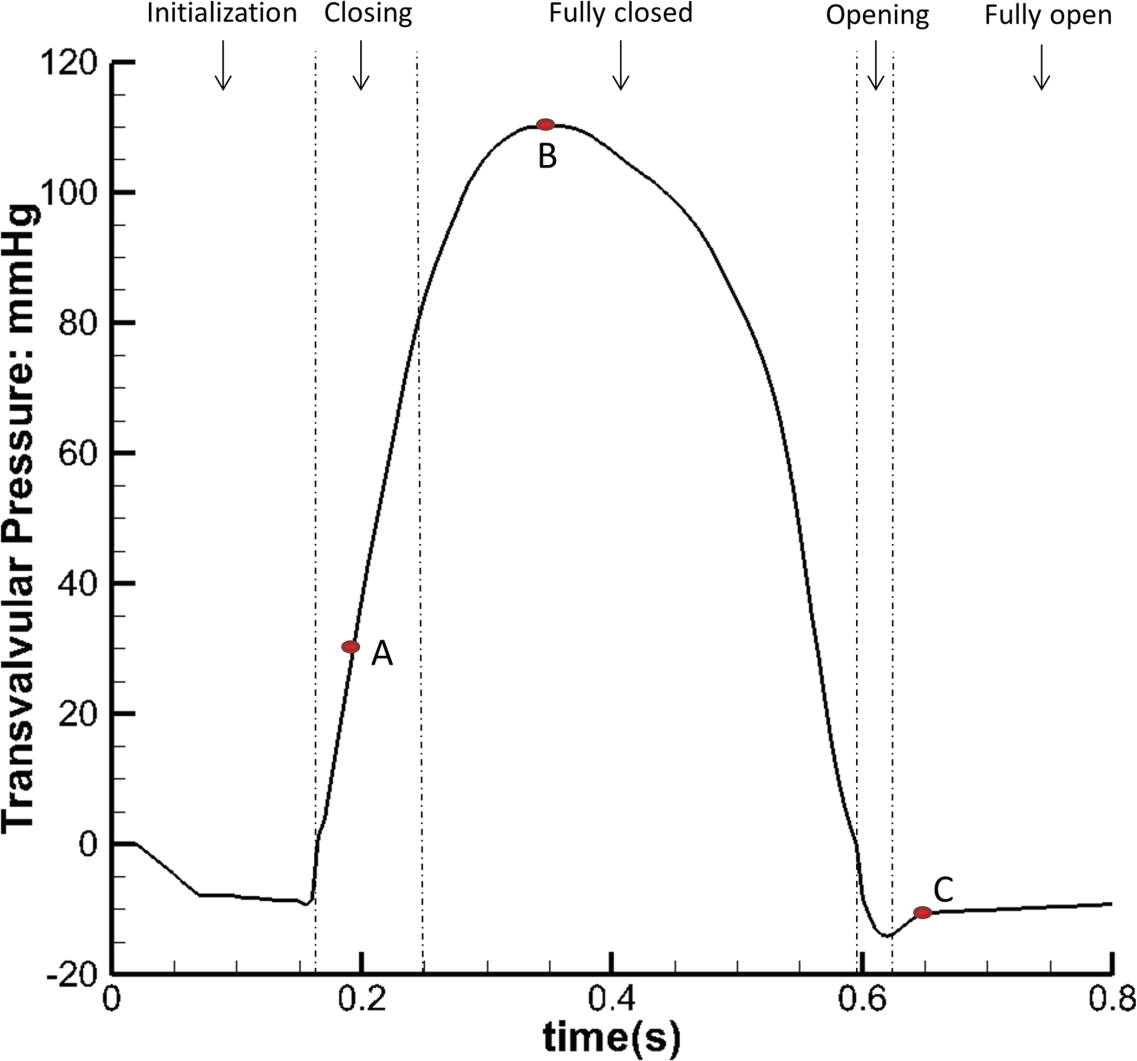
The inlet pressure profile with five phases: initialization, closing, fully closed, opening and fully open phases. We further define three time points: time A is when the MV is closing, time B is when MV is fully closed and under peak pressure (110 mmHg) and time C is when the MV is fully open.

A physiological transvalvular pressure, shown in Fig. 4, is imposed at the inlet, and the outlet pressure is held zero. The modelled cardiac cycle includes the initialization phase, MV closing and fully closed phases, then MV opening and fully open phases. We also choose three time points (identified as times A, B and C as shown in Fig. 4) to perform a detailed analysis. To model the papillary muscle movements, the ends of chordae are constrained with displacement boundary conditions measured from experimental data similar to Wang & Sun (2013).

**Fig. 5.**
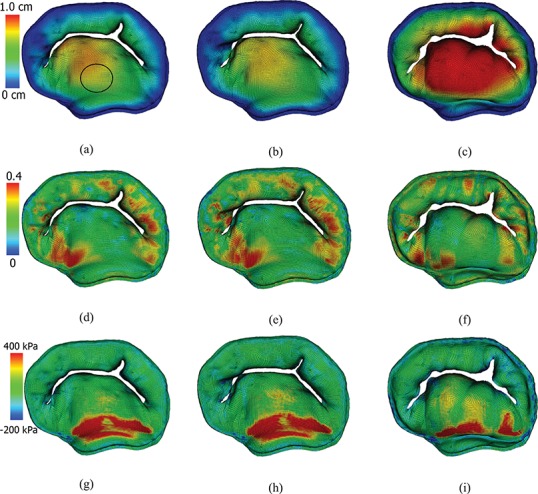
Contours of the closed MV. Top row: by the displacement magnitude for MV models with (a) complex, (b) pseudo-fibre and (c) simplified chordae models. Middle row: the maximum principal strain for MV models with (d) complex, (e) pseudo-fibre and (f) simplified chordae. Bottom row: the maximum principal stress for MV models with (g) complex, (h) pseudo-fibre and (i) simplified chordae. See online version for colours.

The IB/FE scheme used in this study is implemented in the open-source IBAMR software (https://ibamr.github.io), which is an adaptive and distributed memory parallel implementation of the immersed boundary method leveraging several open-source computational frameworks, including SAMRAI (https://computation.llnl.gov/projects/samrai), PETSc (https://www.mcs.anl.gov/petsc), libMesh (http://libmesh.github.io) and hypre (http://www.llnl.gov/casc/hypre) to perform core functionality. The full simulations are run on the supercomputing services ARCHER (http://www.archer.ac.uk) and ARCHIE-WeSt (http://www.archie-west.ac.uk). Some early tests were also conducted at the Barcelona Supercomputing Centre (https://www.bsc.es). A typical simulation of one cycle using 32 processors takes about 9 days in wall clock time on ARCHER or ARCHIE-WeSt.

## 6. Results

### 6.1 Structural deformation and fluid dynamics

**Fig. 6.**
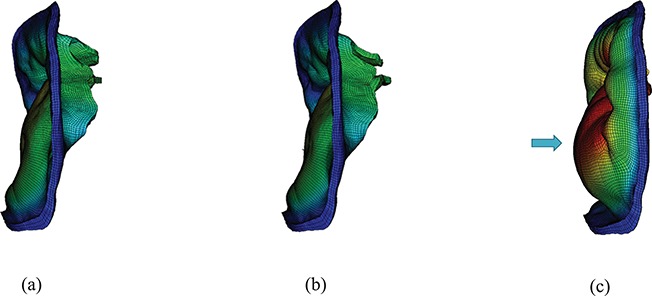
Side view of the three models at time B: (a) the complex model, (b) the pseudo-fibre model and (c) the simplified model. Notice the substantial bulge in the anterior leaflet in (c) shown by an arrow.

**Fig. 7.**
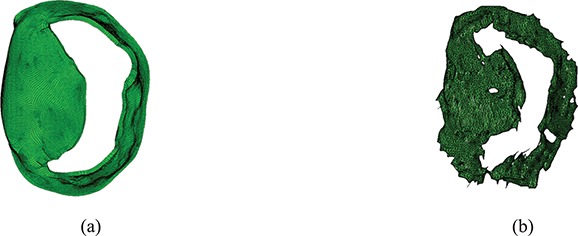
Comparison of leaflet shapes at MV opening: (a) the complex model at time 0.615 s and (b) the segmented MV data from CT scan.

**Fig. 8.**
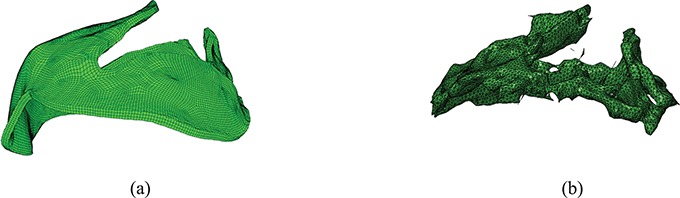
Comparison of leaflet shapes at MV opening: (a) the complex model at time 0.615 s and (b) the segmented MV data from CT scan.

Figure 5 shows the deformation and the maximum principal strain and stress of the three MV models at time point B. Figure 5(a and b) shows that the complex and pseudo-fibre models generate similar closed valve configurations, but the simplified chordae model (Fig. 5(c)) is quite different. The maximum displacement of the MV leaflets for complex and pseudo-fibre models is 0.85 cm and 0.80 cm, respectively. However, the anterior leaflet belly of the simplified model has an unrealistic large bulging into the left atrium, with a maximum displacement of 1.24 cm, which is shown in Fig. 6(c). All three models have a visible gap, which results from the regularization of the delta function (Gao *et al.*, 2014a; Griffith *et al.*, 2009; Ma *et al.*, 2013); however, this gap is much larger in the simplified model, as shown in Fig. 5. The ‘finger-like’ bumps in this case is resulted from the fact that the chordae run through the leaflets. In addition, MV orifice shape is compared between simulated results and segmented data from the same CT images mentioned in Section 3. Because of the image quality, only three phases in diastole can be segmented. Here, we choose the highest quality image and compare it to the complex chordae model. As shown in Figs 7 and 8, the simulated MV leaflets shape matches the CT scan reasonably well despite the fact that the segmented data doesn’t have enough information near the annulus ring, but it captures more details for the rest of leaflets. For example, the bumps near the chordae–leaflet attachment are more pronounced in the segmented data than the simulated data.

In all models, higher stress regions are found in the anterior leaflet belly region, close to the annulus ring (Fig. 5(g–i)), which are in agreement with previous observations (Gao *et al.*, 2014a; Ma *et al.*, 2013). However, notice that the simplified model has higher stress in the anterior leaflet than the other two models. We further compute the averaged maximum principle stress, strain and displacement of a circular region (radius 0.5 cm) in the anterior leaflet (Fig. 5(a)). The results are listed in Table 3. With the chordae stress included, the complex and pseudo-fibre models yield similar results, but the simplified model has a clear discrepancy.

**Table 3:** Averaged maximum principle strain, stress and displacement of the anterior leaflet in a circular region defined in Fig. 5(a)

	Complex chordae	Pseudo-fibre chordae	Simplified chordae
Maximum principal strain	0.177	0.178	0.172
Maximum principal stress (kPa)	148	154	216
Displacement (cm)	0.722	0.697	1.07

**Fig. 9.**
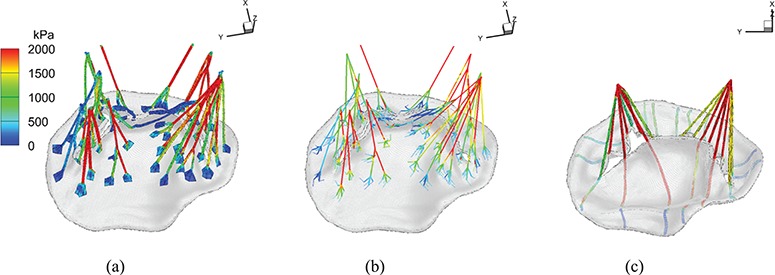
The maximum principal stress at time B: (a) complex model, (b) pseudo-fibre model and (c) simplified model. See online version for colours.

Figure 9(a–c) show the maximum principal stress on chordae for all three models at time point B. The complex and pseudo-fibre models have very similar stress patterns: most of the chordae in the middle experience high stress whereas the chordae attached to commissure area tend to have less stress. For the simplified model, chordae attached to anterior leaflet have high stresses because of the large stretches, and near the leaflet commissures, high stresses also are observed. The average stresses of the chordae attached to the anterolateral and posterolateral papillary muscle groups are 1890 kPa and 2740 kPa, respectively, for the complex model; 2263 kPa and 2914 kPa for the pseudo-fibre model; and 3460 kPa and 3198 kPa for the simplified model. The simplified model has higher values than the other two MV models especially for the anterolateral muscle group.

**Fig. 10.**
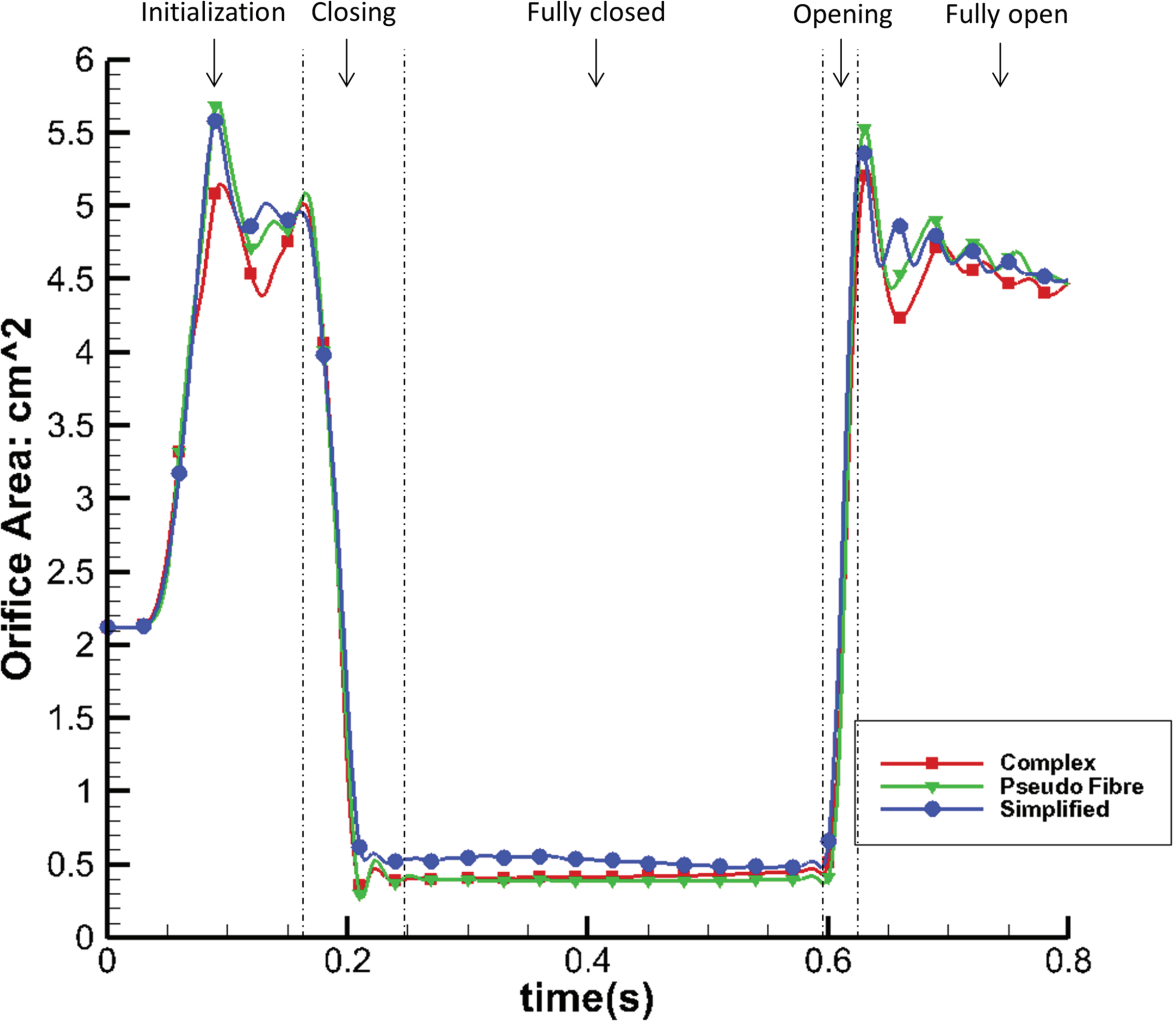
Orifice area change of the three different models.

We also calculate the orifice area for each model by projecting the deformed leaflets onto the $z=0$ plane. The results are plotted in Fig. 10. When the MV is opened at time C, the complex model has a maximum area of 5.21 cm$^2$, while the pseudo-fibre and simplified models have slightly larger areas (maximum: 5.68 cm$^2$ and 5.58 cm$^2$) than the complex model. This is presumably because the pseudo-fibres do not constrain the MV as effectively as the solid chordae, and the simplified model does not have an effective chordae geometry, despite the fact that chordae run through the leaflets. When the MV is closed at time B, both the complex and pseudo-fibre chordae models have smaller orifice areas than the simplified chordae, as shown in Fig. 5. Further, by calculating the time that different models reach the same closure state (e.g. to an orifice area of 0.52 cm$^2$ at 0.165 s), we can show that the complex and pseudo-fibre models have slightly faster MV closure speeds (about 0.022 s faster) than the simplified model, as shown in Table 4.

**Fig. 11.**
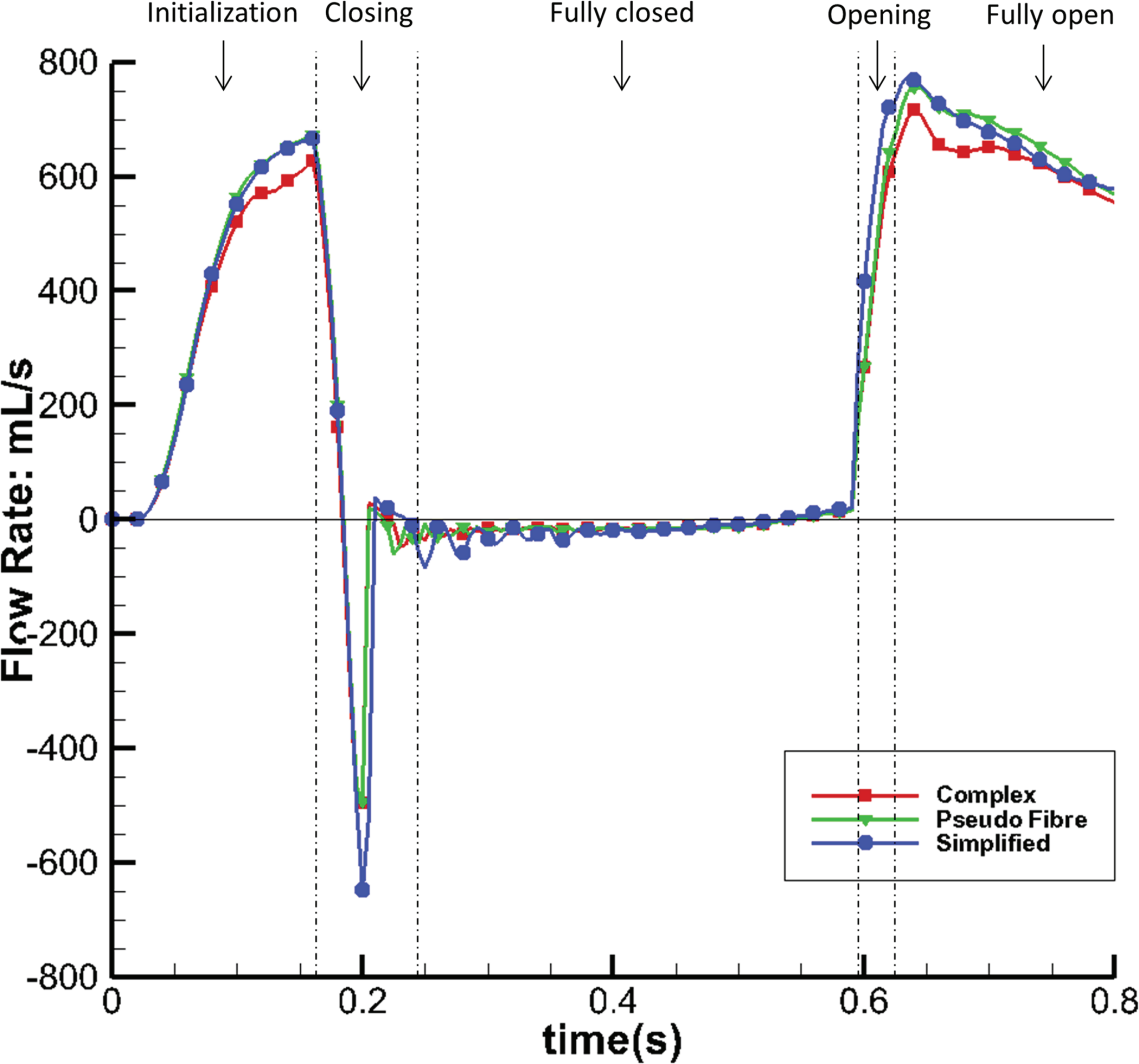
Flow rate through the MV of the three different models.

Figure 11 shows the flow rate through the MV. As suggested by the orifice area changes detailed in Fig. 10, faster MV closure leads to smaller regurgitant flow volumes: 8.66 mL for the complex model and 8.69 mL for the pseudo-fibre model. By contrast, the simplified model has a significantly higher regurgitation volume of 12.81 mL.

**Table 4:** MV orifice and closing speed comparison

	Complex chordae	Pseudo-fibre chordae	Simplified chordae
Orifice area at 0.165 s	4.93 cm$^{2}$	5.00 cm$^{2}$	4.86 cm$^{2}$
Time when orifice area = 0.52 cm$^{2}$	0.208 s	0.208 s	0.230 s

**Fig. 12.**
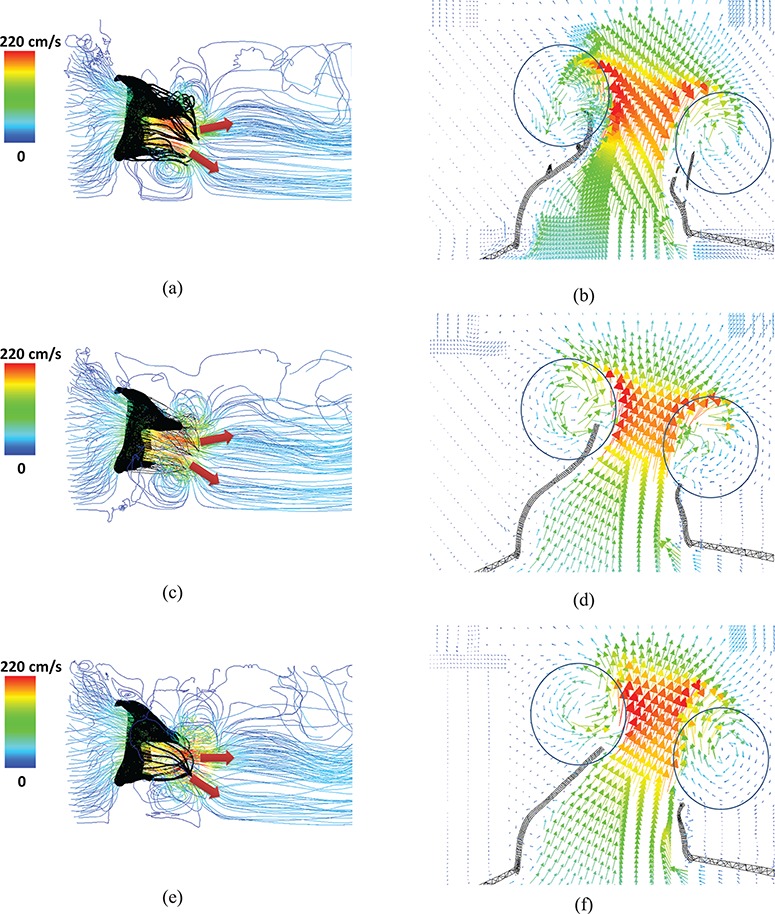
Velocity streamlines and vector field of a cross-section, for the complex model (a and b), the pseudo-fibre model (c and d) and the simplified model (e and f). The two jet directions are shown by arrows, and the vortices around the leaflets can be seen in the circled areas. See online version for colours.

Figure 12 shows the streamlines of the three models at time C when the MV is fully open. Two flow jets are formed as shown by the arrows (Fig. 12(a, c and e)), one close to the anterior leaflet and the other close to the posterior leaflet. The complex and pseudo-fibre models have similar maximum filling velocities (227.1 cm/s and 228.9 cm/s, respectively), and both are lower than that of the simplified model (260 cm/s). Figure 12(b, d and f) shows the corresponding flow vectors around the leaflets. It can be seen that all three models are associated with vortices behind the leaflet tips. Because the pseudo-fibre chordae are modelled as one-dimensional elastic fibres, in the continuum equations, they would not interact with the fluid. In practice, however, interaction between the flow and the chordae is mediated by regularized delta functions, and the finite extent of the delta function kernel imbues the chordae with an effective thickness. Further, at the present mesh resolutions, the Eulerian grid spacing is comparable to the cross-sectional dimensions of the pseudo-fibres. Consequently, from the standpoint of the fluid dynamics, the effective numerical thickness of the pseudo-fibre chordae is similar to that of the volumetric models. Under further grid refinement, it would be necessary to fix the support of the regularized kernel function associated with the pseudo-fibre model, as done in prior work on modelling the interaction of thin rods with fluids using the generalized IB method (Griffith & Lim, 2012; Lim *et al.*, 2008), to provide the pseudo-fibres with a fixed physical thickness.

**Fig. 13.**
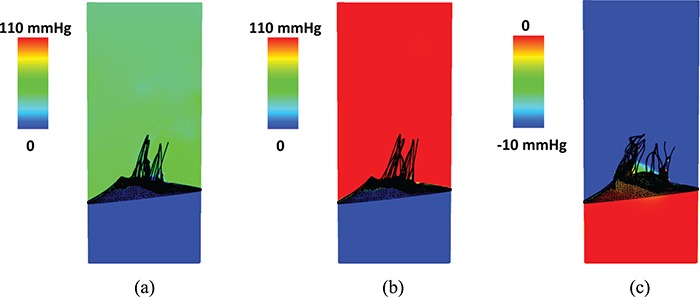
Pressure field for the complex model at (a) time A (closing), (b) time B (peak adverse pressure) and (c) time C (fully open).

Figure 13 shows the pressure fields for the complex model at different time points. The other two models yield similar results.

### 6.2 Energy analysis

**Fig. 14.**
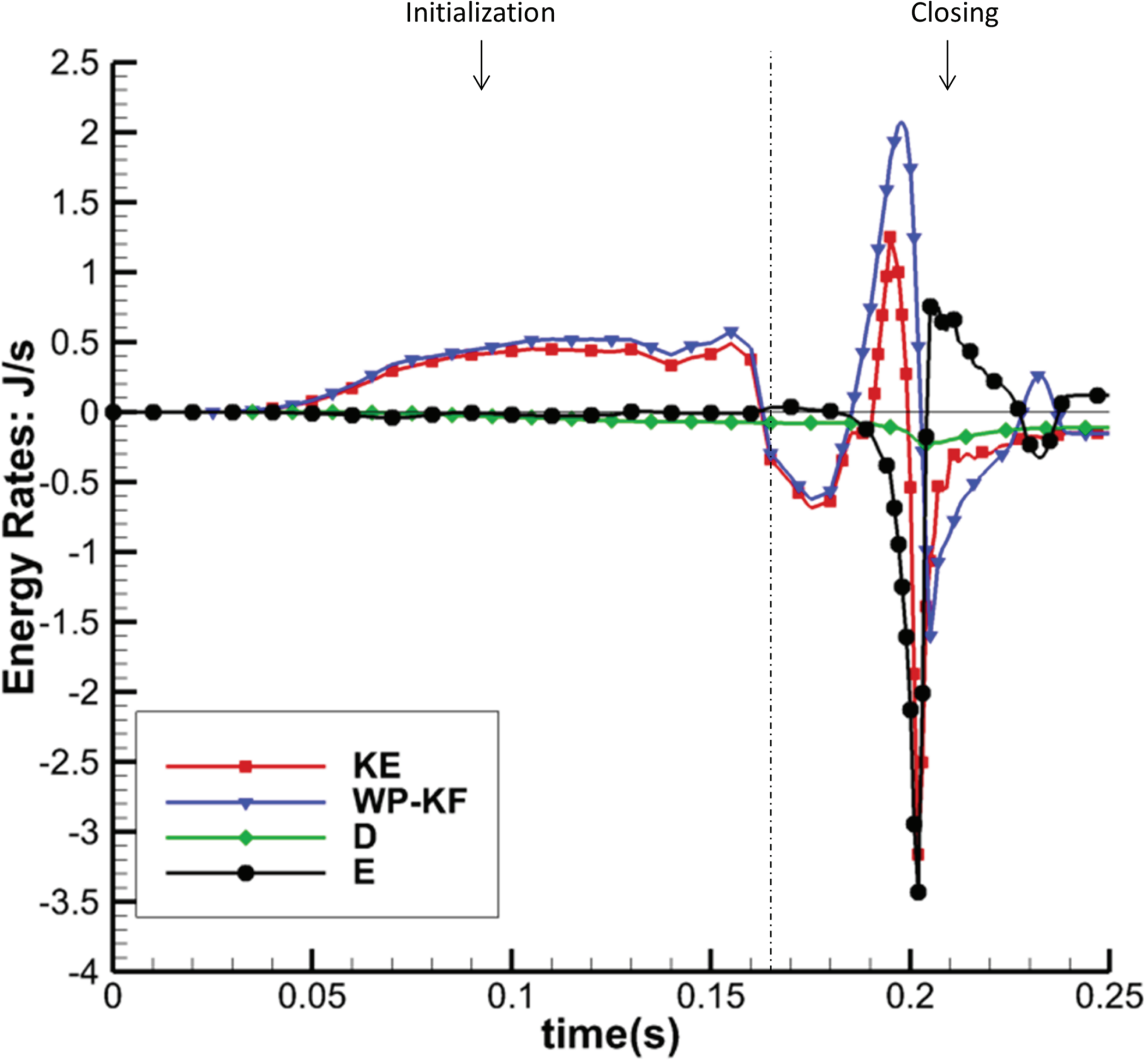
Energy budget for the complex model during the initialization and closing.

**Fig. 15.**
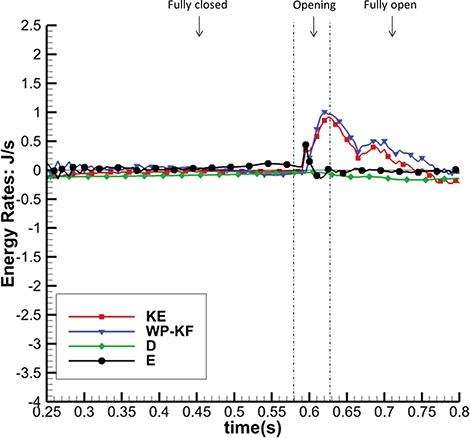
Energy budget for the complex model during fully closed, opening and fully open phases.

To further quantify the dynamics of these different models, we perform an energy analysis. Figures 14 and 15 show the energy budget for the complex model. It is clear that in the fully open phase, kinetic energy dominates the energy distribution and consumes most of the input work (WP-KF). In the closing and opening phases, the MV strain energy (E) starts to have a bigger impact on the energy distribution. The large oscillations in E are associated with the strong motion of the MV during these phases. In the fully closed phase, the coupled system is settling down, and all energy terms gradually decay to zero. Similar energy budget patterns are found in the other two cases. Table 5 summarizes all the energy budget terms at time A (closing) and C (fully open). It shows that 80% of input work is used to increase the kinetic energy at time C and that the MV consumes 94% of the total energy from both the input work and the kinetic energy at time A.

**Fig. 16.**
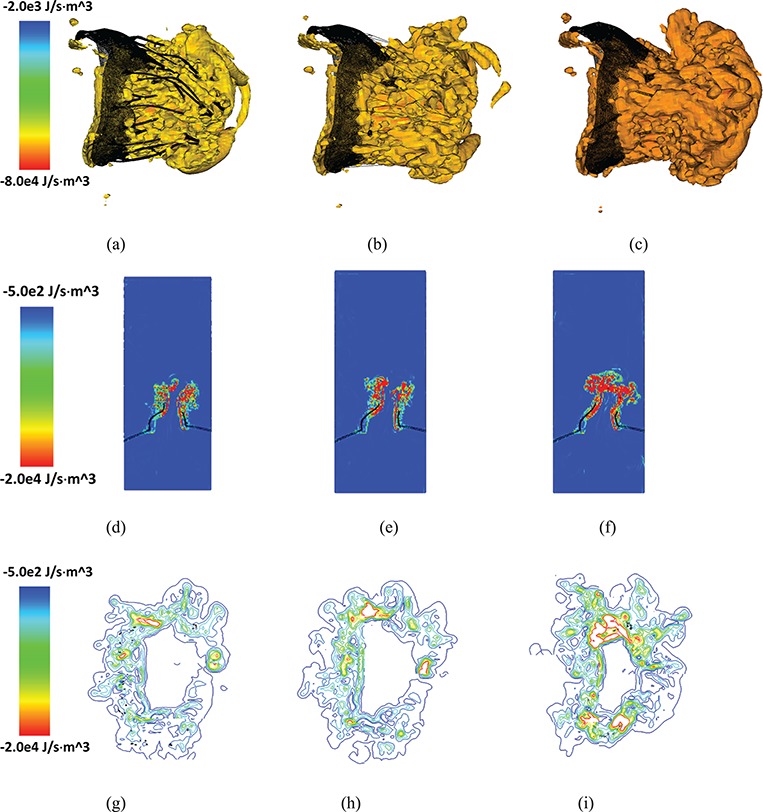
Isovolume plots for energy dissipation function ($\varPhi $) for (a) the complex model, (b) pseudo-fibre model and (c) simplified model. The corresponding 2D sliced views and contour plots around the chordal cross-sections of $\varPhi $ are shown in (d, e and f) and (g, h and i), respectively. See online version for colours.

We further define the energy dissipation function,

(22) \begin{equation*} \varPhi=-\mu (\nabla \mathbf u+\nabla \mathbf u ^{T}): \nabla \mathbf u, \end{equation*}

and plot the dissipation field for the MV models at time C (fully open) in Fig. 16. We can see that high dissipation exists close to the MV leaflets and chordae structure. Energy is also dissipated at a higher rate close to the atrial side of the leaflet because of the high shear stress. Figure 16(g–i) shows a scliced view of the dissipation field associated with the chordae. Overall, the complex and pseudo-fibre models have similar energy dissipation patterns near the chordae structure. However, the simplified model seems to cause a greater dissipation when the MV is opening.

**Fig. 17.**
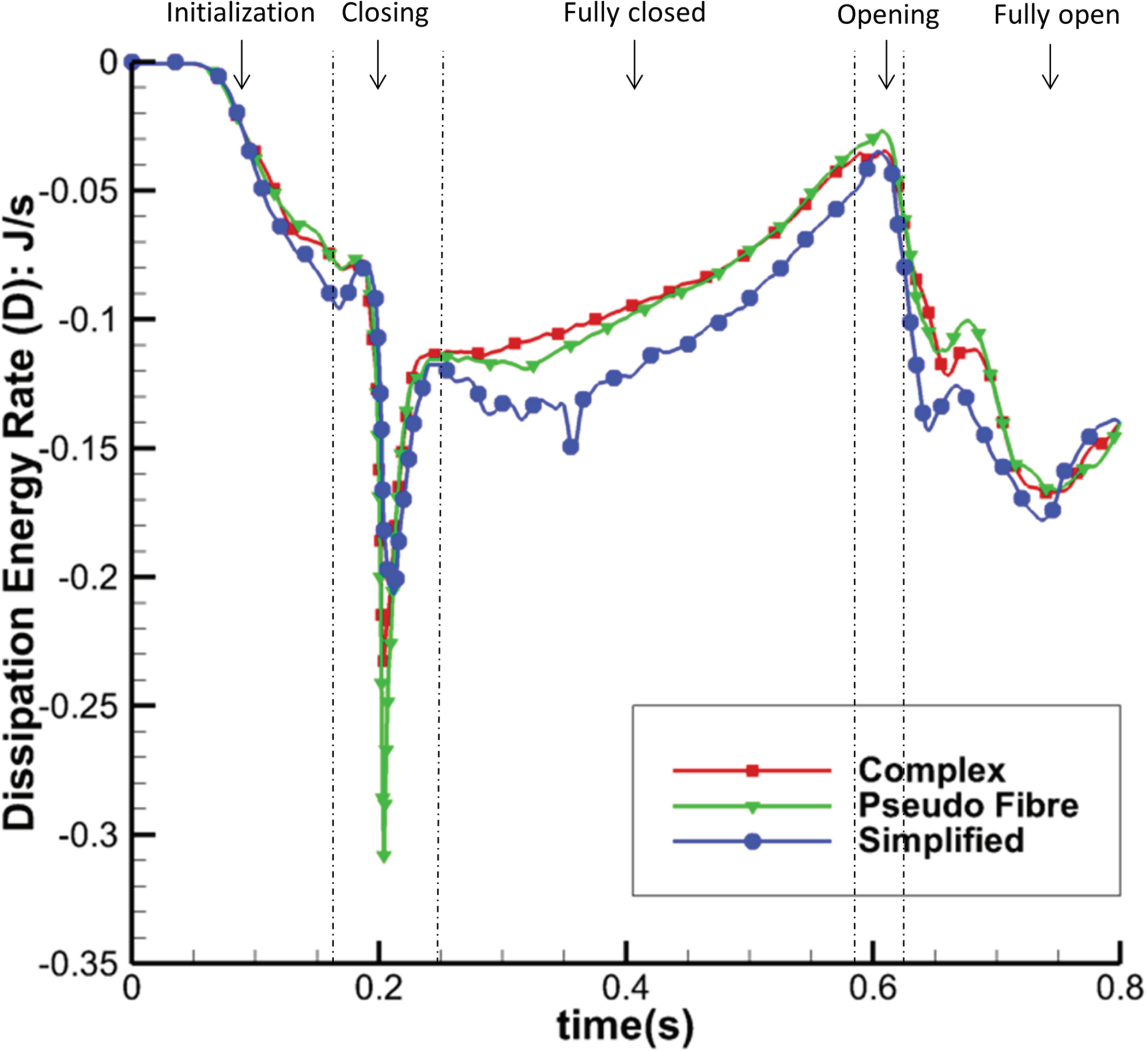
Dissipation Energy Rate (D) of the three models.

Figure 17 shows the total energy dissipation rate $D$ against time. When the MV is closing or opening, D increases, as to be expected. Again, the complex and pseudo-fibre models have similar patterns, but the simplified model introduces a higher energy dissipation rate. On the other hand, during the MV closure phase, when the inlet pressure increases quickly, the pseudo-fibre model has the highest dissipation rate ($-0.31$ J/s), compared to the complex and simplified ones, which are $-0.23$ J/s and $-0.21$ J/s, respectively.

**Fig. 18.**
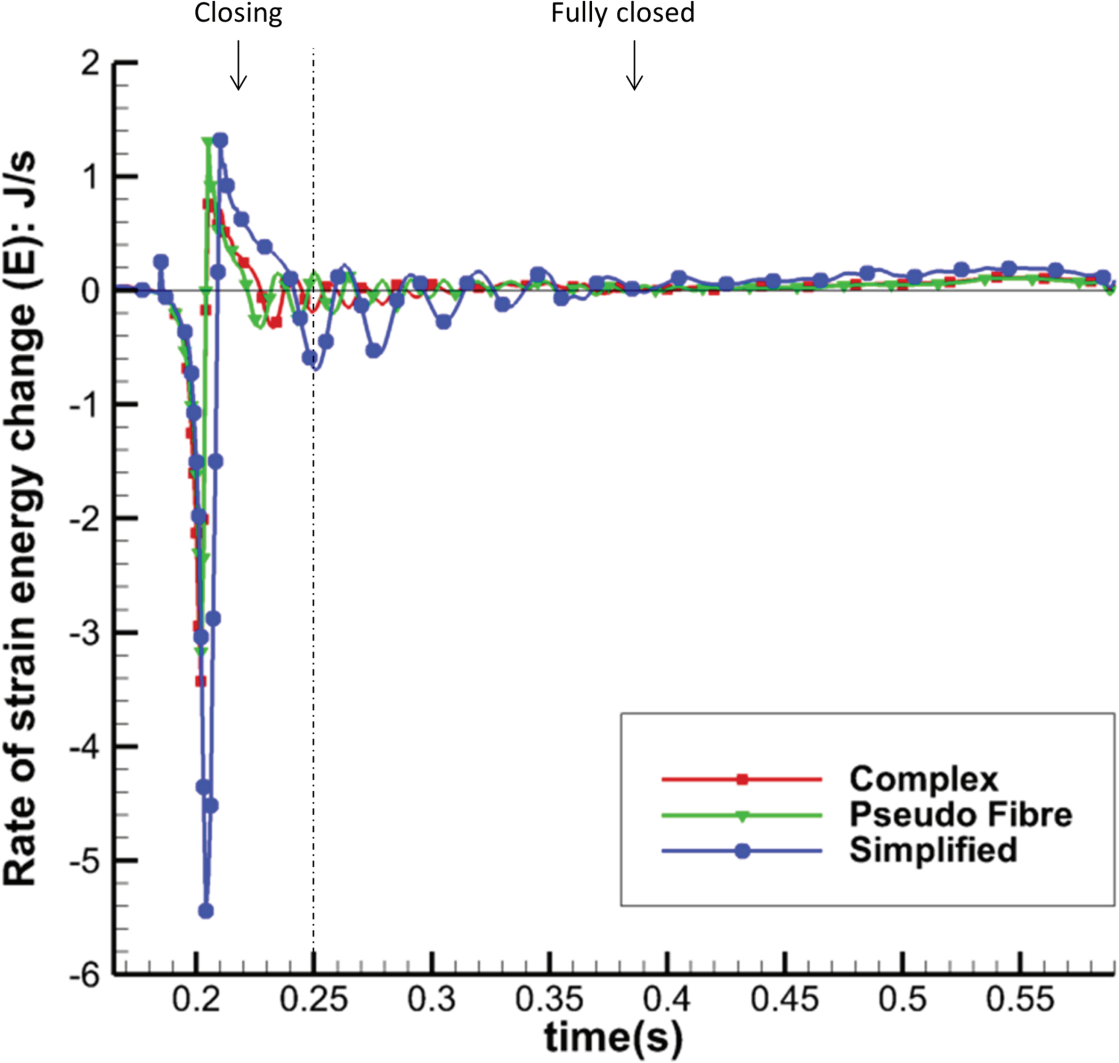
Rate of Strain Energy Change (E) in MV closing and fully closed phases of the three models.

Figure 18 plots the rate of MV strain energy (E) for the three MV models in MV closing and fully closed phases; results in other phases are similar in all the models. It can be seen that the complex and pseudo-fibre models have similar patterns overall, with negative peaks of $-3.43$ J/s and $-3.17$ J/s, respectively, when the MV is closing. However, the simplified model leads to much greater oscillations and a negative peak of $-5.44$ J/s. This is consistent to the fact that simplified chordae model undergoes a larger structural deformation, as is reflected in the displacement contours in Fig. 5(c).

**Fig. 19.**
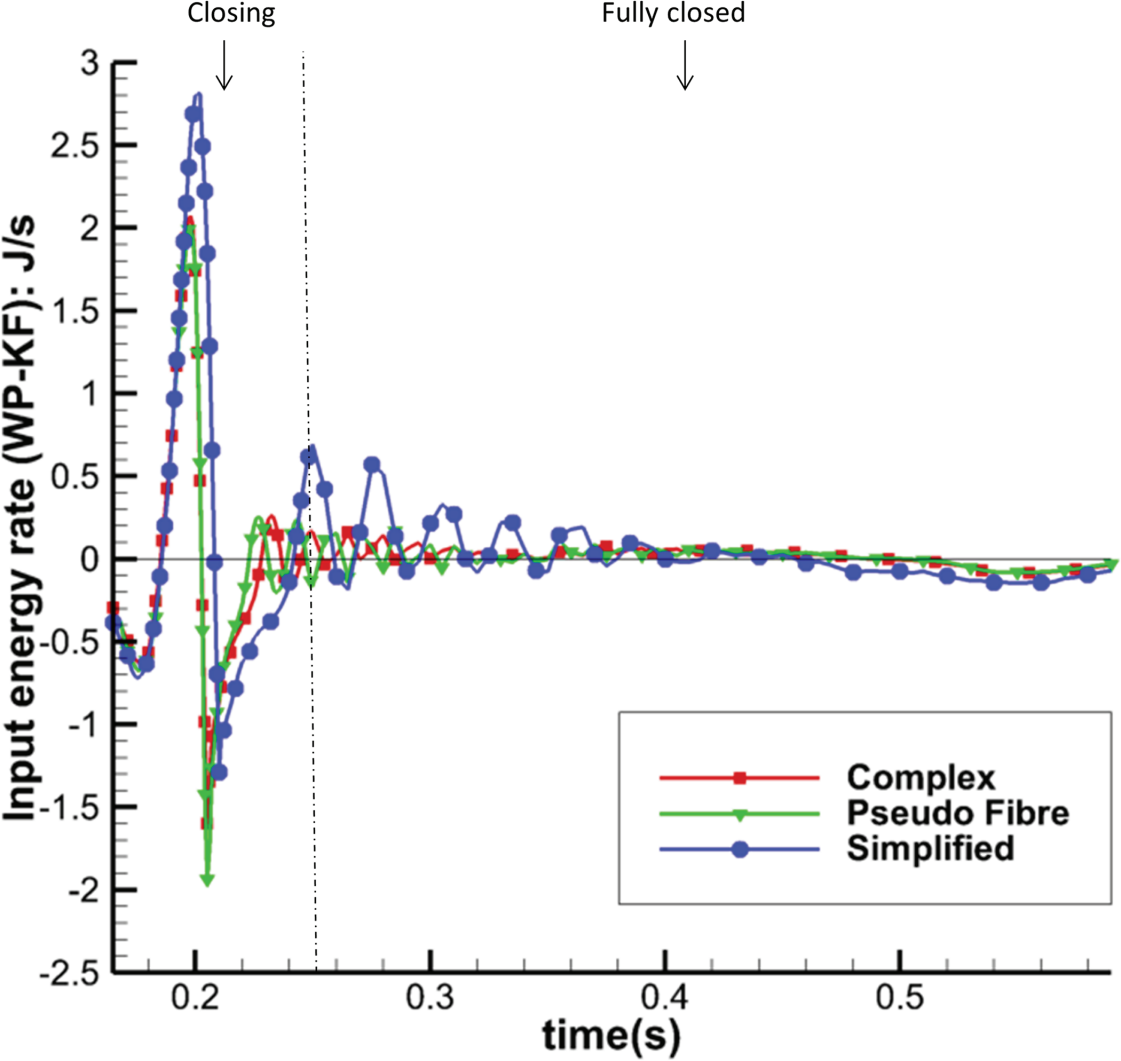
Total Input Energy Rate (WP-KF) in MV closing and fully closed phases of the three models.

Figure 19 shows the input energy rate for the three models in the closing and fully closed phases; results in other phases are similar in all the models. As in Fig. 18, the complex and pseudo-fibre models have similar trends with maximum magnitudes: 2.07 J/s and 2.01 J/s, whereas the input energy rate of the simplified model is much greater (2.81 J/s). In other words, the simplified model requires a greater input energy compared to the other two MV models during the cardiac cycle.

**Table 5:** Energy budget (in J/s) for the complex model at times A and C

	WP-KF	KE	D	E
A (MV Closing)	1.82	-0.38	-0.153	-2.06
			(6% of WP-KF-KE)	(94% of WP-KF-KE)
C (MV Fully Open)	0.759	0.608	-0.0975	-0.0532
		(80% of WP-KF)	(13% ofWP-KF)	(7.0% of WP-KF)

**Table 6:** Integrated energy distribution (in J) over the MV closing phase

	WP-KF	KE	D	E
Complex chordae	-0.00440	-0.0264	-0.0105	-0.00830
	(17% of KE)		(40% of KE)	(43% of KE)
Pseudo-fibre chordae	-0.00384	-0.0239	-0.0109	-0.00942
	(16% of KE)		(46% of KE)	(38% of KE)
Simplified chordae	0.00530	-0.0220	-0.0107	-0.0167
			(40% of KE-WP+KF)	(60% of KE-WP+KF)

The model behaviours can also be seen in the integrated energy budgets. Table 6 shows the energy budgets integration during the period when the MV is closing (from 0.165 to 0.25 s). The complex and pseudo-fibre models produce similar results. Part of the fluid kinetic energy is dissipated due to viscosity (40 and 46%), and the rest is transferred into the MV strain energy (43 and 38%) and to overcome the pressure loading (17 and 16%). However, the simplified model shows a totally different energy distribution: much more strain energy (60% of KE-P-F) is gained from not only the kinetic energy but also the external input energy. Compared to other two models, it also stores about twice as much strain energy during MV closing phase and requires more input energy.

## 7. Discussion

This paper describes an FSI model of the MV that uses an anatomically and physiologically realistic description of the MV leaflets and chordae tendineae that is based on an IB method with FE elasticity (Griffith & Luo, 2017). Our model is verified by comparing the structural results between the IB/FE model and a structure-only model implemented using ABAQUS. In this study, several advances have been made compared with our previous studies (Gao *et al.*, 2014a; Ma *et al.*, 2013): (1) we use physiologically detailed MV leaflets and chordae structures that are based on CT image data, (2) we compared three different chordae structure models and their effects on the MV function and (3) the FSI simulation of the MV dynamics also allows us to conduct an energy budget analysis of the dynamic MV and the flow for the first time.

A realistic MV geometry is necessary for modelling MV dynamics, but it is almost impossible to reconstruct the chordae structure from low-resolution imaging data. Consequently, simplified MV models are widely used (Gao *et al.*, 2014a; Kunzelman *et al.*, 1997; Ma *et al.*, 2013; Prot & Skallerud, 2009; Stevanella *et al.*, 2009). However, researchers started to notice the limitation of such simplifications. For example, Wang & Sun (2013), using a purely structural model, showed that simplified chordae geometry leads to billowing leaflets and incomplete MV closure compared with complex chordae structures. With the help of advanced imaging techniques, more realistic chordae structures are now being reported (Lee *et al.*, 2013, 2015; Toma *et al.*, 2016; Wang & Sun, 2013). Our work goes further to compare the effects of three different chordae structure modelling approaches on overall MV function. In particular, because the chordae structure has often been represented by connections of elastic springs (Griffith *et al.*, 2009; Luo *et al.*, 2012; Ma *et al.*, 2013) when simulating MV dynamics using immersed boundary method, it is interesting to compare the difference between the volumetric chordae and the pseudo-fibre-type representation.

The most notable result of this study is that the complex and pseudo-fibre chordae produce very similar dynamics and kinematics. However, the simplified chordae result in poorer leaflet coaptation and unrealistic bulging in the anterior leaflet, which is also seen in other MV modelling work (Skallerud *et al.*, 2011; Stevanella *et al.*, 2011). Further, compared to the simplified model, the complex and pseudo-fibre models tend to have a better performance in preventing the regurgitation, which can be seen from the orifice area profile (Fig. 10) and the volumetric flow rate (Fig. 11). It is also interesting to note that all of our MV models do not show prolapse, because of the realistic hyperelastic constitutive law used for the leaflets and volumetric structure model which automatically includes the bending stiffness. This is in agreement with our previous study (Luo *et al.*, 2012), which showed that bending in the MV leaflets plays an important role. The complex and pseudo-fibre models also show consistent results with other published results of MV studies. Wang & Sun (2013) reported the average maximum principal stress is approximately 160 kPa on a midsection of anterior leaflet at the systolic peak. Kunzelman *et al*. (2007) used an FE model to find that the maximum principal stress on the anterior leaflet was 254 kPa with a transvalvular pressure of 98 mmHg. Gao *et al.* (2014a) also reported the peak average stress is 115 and 169 kPa, along the radial and circumferential directions, in the anterior belly region. These results are comparable to our model predictions, as shown in Table 3.

The majority of MV modelling research focuses on the mechanics of the MV or the hemodynamics inside the left ventricle (Gao *et al.*, 2014a, 2017b; Wang & Sun, 2013), and comparatively few reported studies have evaluated MV function from an energetic perspective (Choi *et al.*, 2014). Akins *et al*. (2008) pointed out that evaluating energy loss during the cardiac cycle can help assess the impact of valve performance on cardiac function. Choi *et al.* (2014) also attempted to calculate the energy dissipation in a simplified LV-MV model to study the MV performance. Using the energy budget analyses reported in (10)–(17), our work is the first effort to consider the whole cycle of the realistic MV model and FSI from an energetic perspective. This energy analysis reveals that when the MV is fully open, most of the input energy from left atrial contraction is used to increase the kinetic energy, which is then used to pump blood into the left ventricle from the left atrium. However, when the MV is closing or opening, the MV leaflet elastic energy becomes dominant.

The energy distributions also show that the chordae structure plays an important role. In our simulations, even with different mesh elements, the complex chordae and pseudo-fibre chordae lead to very similar energy results, as seen in Figs 16–19. Moreover, Table 6 shows that during early LV systolic phase until the time when the aortic valve opens, most of the kinetic energy is reduced via viscous dissipation and increases in the MV strain energy. However, the simplified chordae introduce more energy exchanges overall, especially when the MV is closing or opening, which can be seen from the larger magnitude peaks in Figs 18 and 19. Further, Table 6 shows that much more strain energy is stored in the simplified chordae, which may be partially responsible for the unrealistic bulging. As a result, the input energy from left ventricular contraction is also substantially increased in this model. The slower MV closing process in the simplified model also leads to a sign change in the input energy contribution in Table 6, indicating a different energy distribution from the other two models.

One of the most important aspect in cardiac energy analysis is to see how energy loss (from viscosity, turbulence and flow separation) affect cardiac function, especially under pathological conditions. Although the energy dissipation term tends to have much smaller magnitude compared with other energy terms, this is the irreversible loss of the system and is thus important over the longer term. Figure 16 gives quantified description of energy loss for the fluid fields of different models. A higher dissipation rate is always found near the structure because of the high shear, particularly during opening. The simplified model shows a different energy dissipation pattern and leads to greater energy dissipation compared to other two models, which suggests decreased efficiency.

This study has several limitations. One of these is that homogeneous material properties are assumed for MV leaflets. Some studies (Kunzelman & Cochran, 1992; May-Newman & Yin, 1998) have suggested that this is not realistic, and regionally dependent material parameters should be considered in future works. Although we don’t have sufficient experimental data at hand to directly compare with our CT-scan-based MV models at additional time points in the cardiac cycle, our results are consistent with published results of MV studies. Finally, we have shown that the energy budget analysis for the complex flow field has a great potential to provide useful pathological indicators; however, additional critical analyses will be required before it can be used for physical insights into differences between healthy and pathological cases. This requires extensive evaluation on how the energy budget is affected by sufficient number of pathological cases. Finally, we have not attempted to study pathological changes either in the chordae structure or the MV leaflets. However, with the framework developed, it is our future aim to model various sceneries with clinical significance.

## 8. Conclusions

We have studied the effects of different chordae structures in a dynamic MV model that includes a detailed description of the valve leaflets and chordae. Our computational approach includes FSI, anisotropic hyperelastic constitutive modelling and energy budget analysis. The simulation has been carefully verified against the commercial software ABAQUS under static loading conditions. Our results show that detailed chordae structure is superior than our previously used simplified chordae structure, which, even when modelled as three-dimensional structures and embedded through the leaflets, leads to poor leaflets coaptation, large bulging on the anterior leaflet belly and different energy-transferring behaviour. On the other hand, the pseudo-fibre representation is a good approximation of the more realistic three-dimensional complex solid chordae construction. Indeed, similar MV closure shape, strain and stress patterns and energy distribution patterns are observed using either the complex or the pseudo-fibre models. This suggests that pseudo-fibres may be a reasonable choice to model mitral chordae in future.

## Figures and Tables

**Fig. A1.**
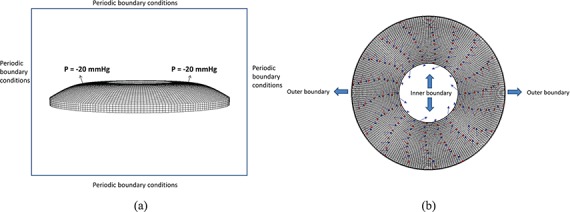
The disc geometry (a) side view, (b) top view.

**Fig. A2.**
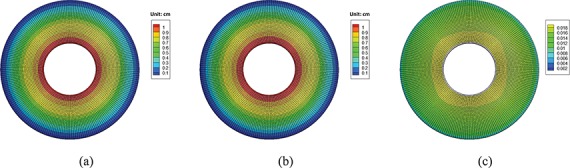
Normed displacement plot from (a) IB/FE and (b) ABAQUS. (c) The relative difference (only showing the regions below 2$\%$).

**Fig. A3.**
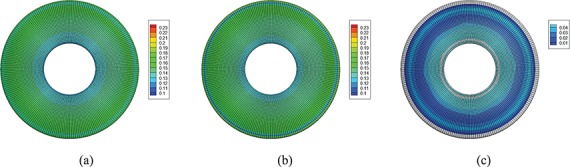
The logarithmic strain generated by (a) IB/FE and (b) ABAQUS. (c) The relative difference (only showing the regions below $5\%$).

**Fig. B1.**
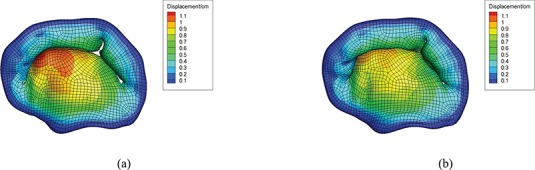
Displacement plot from (a) IB/FE and (b) ABAQUS. See online version for colours.

## Appendix. A. Verification test 1: deformation of a circular disc under constant pressure

Model verification is first done on a simple axis symmetric geometry, as shown in Fig. A1(a and b). It is a disc-shaped structure with radius of $0.4$ cm and thickness of $0.03$ cm. In the IB/FE model, the disc is immersed in a viscous fluid with density of 1 g$\cdot $cm$^{-3}$ and dynamic viscosity of 1 g$.\cdot $cm$^{-1}\cdot $s$^{-1}$.

A constant pressure of $-20$ mmHg is applied on the top surface of the structure in both cases, and in the IB/FE model, periodic conditions are applied on the boundaries of the computational domain $\varOmega $. Equation (18) is used for describing the disc material property. Two families of fibres are defined simply along the circumferential direction and radial direction, respectively.

Figure A2(a and b) show the distribution of displacement magnitudes obtained from IB/FE and ABAQUS. The maximum displacement norm is $11.11\,\textrm{mm}$ in the IB/FE model, which is slightly less than the value in the ABAQUS model that is $11.13~\textrm{mm}$. It is clear that two plots have almost the same patterns.

The relative difference in the displacements is computed via

(A.1) \begin{equation*} \textrm{relative difference}=\frac{\textrm{norm of displacement difference}}{\textrm{average displacement norm from Abaqus}}\times 100\%. \end{equation*}

Figure A2(c) shows regions that have relative difference less than $2\%$, except for the inner edge; the relative difference is very small in most of the disc.

Figure A3(a and b) shows the logarithmic maximum principal strain. The maximum values are 0.2143 in the IB/FE model and 0.2485 in the ABAQUS model. Using the same relative difference definition given by (A.1), Fig. A3(c) shows all the nodes with relative difference below $5\%$.

## Appendix. B. Verification test 2: deformation of MV under constant pressure

We use the MV with complex chordae structure to compare the IB/FE and the ABAQUS models under static loading conditions. In the IB/FE model, the MV is immersed in a 5 cm$\, \times \,$4 cm$\, \times \,$6 cm box filled with the same viscous fluid as in the disc case. A constant pressure of $-30$ mmHg is applied on the atrial side of the leaflets, the annulus ring is fixed by using stiff tethering forces and displacement boundary conditions are applied to the chordae ends. Periodic boundary conditions are applied to boundaries of the computational domain $\varOmega $. The same structural constraints and loading conditions are also used in the ABAQUS model. Because of the large deformation in MV model, dynamic explicit analysis in ABAQUS is used. Equation (18) is used for the leaflets, but a Neo-Hookean material is used for the chordae structure

(B.1) \begin{equation*} W=C(I_{1}-3), \end{equation*}

with $C= 740$ kPa.

Figure B1(a and b) shows the distribution of displacement magnitudes from the IB/FE and the ABAQUS models when the MV reaches an equilibrium state by maintaining constant loading for a long time. The maximum values are 1.1372 cm in the IB/FE model and 1.0847 cm in the ABAQUS model. Both models give good agreement in the MV closure shapes, with a mean difference of 0.0689 cm, except for slightly bigger gaps in the IB/FE model as shown in Fig. B1(a) due to the Eulerian mesh density and the use of delta function in the FSI model.
